# Notes from Our Scrap-Book

**Published:** 1881-12

**Authors:** 


### NOTES FROM OUR SCRAP-BOOK.

These few notes of drugs having the symptom of *weakness*, etc., *in abdomen or stomach*, have been collected from time to time and are, I think, reliable and may prove of use.

*Am. Carb.*—Empty feeling in stomach; fullness after eating.

*Am. M.*—Empty or hungry feeling: sensation as if from fasting yet fullness in stomach, worse after breakfast.

*Anac.*—First sensation of fasting in pit of stomach, then pressure in stomach.

*Ant. Crud.*—Sensation of emptiness in abdomen going off after meals.

*Bapt.*—Sinking, gone feeling, fainting; tongue brown.

*Bell.*—Emptiness in stomach.

*Bry.*—Feeling of emptiness in stomach with distension of abdomen.

*Calc. Phos.*—Empty sinking around navel or belly.

*Carbo. An.*—Faint, gone feeling, *not* relieved by eating.

*Cocculus*—Emptiness and hollowness in abdomen.

*Digit.*—Weakness, sinking as if he were dying.

*Gels.*—Feeling of emptiness and weakness in stomach and bowels.

*Glon.*—Faint feeling at pit of stomach.

*Hydras.*—Faintness at stomach, *goneness, sinking*, with violent palpitation of heart.

*Ignat.*—Weakness, sinking in pit of stomach.

*Ipecac.*—Stomach feels relaxed, hanging down.

*Lilium*—Hollow, empty sensation in stomach and bowels.

*Mag. M.*—Nausea, followed by weakness and fainting and coldness in stomach.

*Meph.*—Nausea with emptiness in stomach.

*Merc. Iod.* (F.)—Nausea with emptiness in stomach and weakness.

*Mur. Ac.*—Empty sensation extending into abdomen, no hunger; from 10 A. M. to evening.

*Natr. M.*—Great weakness by spells.

*Nicco.*—Sensation of emptiness without hunger.

*Petroleum*—Sensation of emptiness and weakness in stomach. Gastralgia better from eating.

*Phos.*—Sensation of emptiness in abdomen which seems to aggravate other complaints.

*Plumb.*—Nausea, weakness.

*Sanguin.*—Sensation of emptiness *soon* after eating.

*Sepia*—Painful sensation of emptiness in stomach and abdomen, worse when thinking of food.

*Stannum*—Sinking, gone feeling.

*Staphis.*—Weakness in abdomen as if it would drop down, wants to hold it up (*Agnus* C.). E. J. L.

(TO BE CONTINUED.)

